# Three-wave longitudinal prediction of positive mental health in Germany and China

**DOI:** 10.1371/journal.pone.0287012

**Published:** 2023-12-21

**Authors:** Jürgen Margraf, Kristen L. Lavallee, Xiao Chi Zhang, Silvia Schneider

**Affiliations:** Mental Health Research and Treatment Center, Ruhr-Universität Bochum, Bochum, Germany; National Cheng Kung University College of Medicine, TAIWAN

## Abstract

The present study is a follow-up of a prior study examining a broad range of longitudinal predictors of dimensional positive mental health (PMH) and negative mental health (NMH), using cross-cultural data from the Bochum Optimism and Mental Health (BOOM) study. The present study sought to expand prior findings on positive mental health predictors to a longer longitudinal timeframe. The analysis, specifically, tests our prior model using a third time point, The following positive constructs were examined in relation to later positive mental health: resilience, social support, social rhythm, family affluence, physical health and expectations for fertility. Negative predictors depression, anxiety, and stress were also examined in relation to time 3 positive mental health. Participants included university student samples from Germany (N = 591) and China (N = 8,831). Structural equation modeling was used to examine the effects of predictors on mental health. In China, three of the six salutogenic predictors (social rhythm regularity, positive mental health, resilience) at baseline were predictive of positive mental health at both follow-ups with generally small, but significant effects. Social support at baseline predicted more, and stress and anxiety predicted less positive mental health at follow-up 1, with generally small effects. Depression at baseline predicted less positive mental health at follow-up 2. In Germany, two of the six salutogenic predictors (positive mental health, social support) at baseline were predictive of positive mental health at both follow-ups, with generally small effects. Pathogenic predictors were not predictive of positive mental health at either follow-up. According to multi group analysis, the paths from positive mental health baseline to positive mental health follow-up 1 (FU1) as well as the path positive mental health FU1 to positive mental health follow-up 2 (FU2) were found to differ between Germany and China. All other paths could be seen as equivalent in Germany and China. Results indicate prediction of positive mental health over an extended period of time, and in particular by salutogenic predictors. Pathogenic predictors were also (negatively) predictive of PMH, but with more mixed results, underscoring the differential prediction of PMH from salutogenic and pathogenic factors.

## Introduction

Increasingly, positive mental health is recognized as a uniquely important facet of mental health, with effects that are distinct from those of negative mental health [1,2]. Positive mental health (PMH) is not defined as the absence of disorder, but rather as a constellation of aspects of flourishing and well-being, such as resilience, and life satisfact, while negative mental health refers to negative mental states that in excess lead to disorder, such as depression and anxiety. Positive and negative mental health are interdependent, yet separate dimensions that are not necessarily mutually exclusive, and can indeed present simultaneously [1–3]. Recently, as part of the BOOM studies (Bochum Optimism and Mental Health) [4], we conducted a longitudinal and multinational examination of the differential effects of positive and negative mental health, demonstrating that over a two-wave time period, salutogenic factors (positive factors anticipated to contribute to well-being) predicted both positive and negative mental health (positively for PMH and negatively for negative mental health) [4]. Pathogenic predictors (negative predictors anticipated to contribute to a decline in mental health), on the other hand, were primarily related to later negative mental health, but not necessarily to PMH [4]. In addition, these results were consistent cross-culturally, with findings generally holding across Germany, Russia, and China [4]. The present study extends this research by examining these results across an additional, third time point in a study of effects in Germany and China (third time point data not available for Russia). A brief background will be presented here. For expanded background, please cosult the prior work [4].

The examination of both salutogenic and pathogenic factors in predicting mental health is well supported [5,6]. Such factors can include biological factors (such as physical health), psychological factors (such as resilience and life satisfaction) and social factors (such as socioeconomic status and social support) [7–10]. Longitudinal research finds that pathogenic predictors tend to predict later mental disorder (both incidence and relapse), and salutogenic factors tend to predict the remission of those disorders [11]. More longitudinal research is still needed on salutogenic predictors of positive mental health, as this has still tended to be an understudied area. This was the impetus for our previous longitudinal study examining both positive and negative factors contributing to mental health across two time points and three cultures (Germany, Russia, and China) [4]. The reader may refer to Margraf et al. (2020) [4] for the full background on variable selection. Margraf et al. (2020) found that physical health, social support, positive mental health, social rhythm, resilience, and fertility wish (the salutogenic factors in this study) positively predicted positive mental health [4]. Five of the salutogenic factors (resilience excluded) negatively predicted negative mental health (as measured by depression scores) [4]. Anxiety and depression (the pathogenic predictors in this study) were related to increases in depression over time [4]. Anxiety and depression were not, however, predictive of later positive mental health [4]. Stress (another pathogenic predictor) was related to reducted later positive mental health and increased later depression [4]. Despite some country-level differences in the unconstrained models, a model with effects constrained across the three countries also provided a good fit to the data, indicating that results can generally be considered statistically the same across countries [4]. The results lend support to the concept of positive and negative mental health as related, but separate constructs with independent effects on mental health, rather than as two ends of a spectrum [4].

### Present study

The present study is a follow-up to our prior primary outcome study [4] in the the “Bochum Optimism and Mental Health (BOOM) Studies” [12]. The first study sought to examine the predictors of positive and negative mental health over time and in three separate countries [4]. This study sought to expand prior findings on positive mental health predictors to a longer longitudinal timeframe. The present analysis, specifically, tests our prior model using a third time point, expanding the original large-scale, cross-cultural, multi-national, and longitudinal investigation into primarily salutogenic factors contributing to both positive and negative mental health outcomes, as measured by positive mental health and depression. It is an attempt to contribute solidify the findings on the etiology of mental health by extending the analyses to a longer time period. As in the prior study [4], the objective of this study is to examine the utility of the following positive constructs in predicting positive mental health: resilience, social support, social rhythm, family affluence, physical health and expectations for fertility using a structural equation model. A second objective is to examine negative predictors of depression, anxiety, and stress in predicting time 3 PMH in a structural equation model. Recognizing cultural background as having an important influence on mental health [7–10,13], a third objective is to test the equivalence of the predictive value of these factors across the two cultrues of China and Germany, specifically testing structural equation model path constraints. Due to a change in data collection at the third wave, only data from Germany and China are available for the present study. The Russian cooperation partner had no capacity to continue the follow-up studies. Thus for the present study, we have data only from Germany and China with one baseline and two follow-ups. Mental health at time 3 was hypothesized to be predicted by PMH at time 3 was espected to be positively predicted by salutogenic psychological, biological and social factors [3,4,11].

## Method

### Procedure

The present study utilizes data from the BOOM (Bochum Optimism and Mental Health) studies. BOOM is a longitudinal, cross-cultural investigation into a wide range of risk and protective factors contributing to positive and negative mental health [12,13]. Margraf & Schneider [12] provide a study overview. The study was approved by the Ethics Committee of the Faculty of Psychology of the Ruhr-Universität Bochum on May 12, 2011 and renewed on September 2013. Approvals for the German site were communicated to, and accepted by, the participating Chinese Universities. As the data from China were anonymized, no statement by an institutional board/ethics committee was required for China. Data were collected between 2011 and 2016, with participants recruited via the internet (German and Chinese) and paper mailings (Chinese). The period of time between the first and second time points was about 17 months for each participant. The period of time between the second and third time points was about 13 months. In Germany, the baseline data collection took place between 2011-2016 (data were collected in November each year). Follow-up one data were collected between 2012-2017, and follow-up 2 data were collected between 2013-2018. In China, baseline data were collected between September 2012 and December 2013. Follow-up 1 data were collected between September 2013 and November 2014, and follow-up 2 data were collected September 2014 to November 2015.

German students at Ruhr- University- Bochum (RUB) submitted their questionnaire data via online portal. The portal link was sent to all enrolled students at RUB in 2011, and thereafter only sent to freshmen from 2012 to 2016. Students were offered an incentive (they were entered into drawing for a 20 EUR gift certificate or a tablet computer).

In China, participants, mainly freshmen, were recruited during their first study month via an invitation by mail. The response rate was 94.5%. Data were gathered by an online or a paper-pencil questionnaire administered in a group testing session. Participants received 10 RenMinBi (approximately 1.3 EUR) upon returning the questionnaire.

Participants read an informed consent statement, wereby they learned their answers would be collected pseudonymized. A code was used for anonymization, which enabled linking individuals‘ answers to one another across surveys and measures, while maintaining the participant’s anonymity. Only project supervisors maintained access to these codes. Participants provided implicit consent by electing to continue with the surveys after reading the informed consent statement.

### Participants

University student participants with data at baseline, one-year, and two-year follow-ups were included from two countries: Germany (591) and China (8,831).

Germany. The German sample consisted of 8661 (1,719 had data at Follow-Up 1, and 591 had data at Follow-Up 2) student participants recruited from Ruhr University Bochum from 2011 to 2016. Students were assessed via online survey. German students were recruited by an e-mailed invitation with a link leading to an online questionnaire. The link was sent to all students enrolled at Ruhr University Bochum. They were offered an incentive to take part in a drawing for a gift certificate or a tablet computer.

China. As the data were anonymized from the very beginning of data collection, no statement by an institutional board/ethics committee was required to collect data in China. The Chinese sample consisted of 13,581 (12,057 had data at the first two time points, and 8,831 had data at all three time points) university students from the Capital Normal University Beijing, the Hebei United University, Shanghai Normal University, Guizhou Finance and Economics University, and Nanjing University. Participants who took part in all three waves were included for the further analyses. The difference between who took part in all three waves and those only took part in baseline were compared via chi-square test or t-test.

### Measures

#### Overview

As far as possible, established brief standard instruments such as the Depression Anxiety Stress Scales (e.g., DASS 21) were used to measure the constructs of interest. For all questionnaires used in the analysis, validated German versions exist. Chinese versions of the measures were developed when needed, by using the customary translation-back-translation method as recommended [14]. In cases of discrepancies, this procedure was repeated by the study team until complete agreement was achieved. Measures can be grouped according to the overall design of the research program. Table 2 provides an overview as well as correlations.

#### General outcomes

*Positive Mental Health*. The 9-item PMH-scale was developed in order to provide a brief, uni-dimensional and person-centered instrument to assess positive mental health [3]. The concept of positive mental health combines mainly emotional, but also psychological and social aspects of well-being into a single general construct [3]. People who are mentally healthy tend to have stable relationships, view their lives as having purpose and direction, experience more positive affect, and are more likely to be self-accepting [15]. Psychometric testing confirmed the scale to be a unidimensional self-report instrument with high internal consistency, good retest-reliability, scalar invariance across samples and over time, good convergent and discriminant validity as well as sensitivity to therapeutic change in a series samples from very different backgrounds [3]. Participants respond to statements such as *“I am often carefree and in good spirits*, *I enjoy my life*, *I manage well to fulfill my needs*, *I am in good physical and emotional condition“* on a 4-point likert scale ranging from 1 (do not agree) to 4 (agree). Item scores are combined into a sum score with higher scores indicating higher PMH. The measurement invariance of PMH is established as full strong [16]. Cronbach’s alphas at baseline were .914 (Germany), and .891 (China).

#### Predictors

*Depression*, *Anxiety and Stress*. Negative mental health was assessed using the widely- used Depression Anxiety Stress Scales (DASS-21) [17]. This short form of the DASS-42 [18] assesses a broad range of psychological distress symptoms. It is composed of three 7-item subscales for depressive, anxiety and stress symptoms over the past week. The subscales may serve as outcome measures and as screening and monitoring instruments [19–21]. Items are rated on a 4-point likert scale from 0 (did not apply to me at all) to 3 (applied to me very much or most of the time). Responses can be averaged within subscale or across all three for a total item score. Psychometric properties are well established in both clinical and non-clinical samples [17,21] and are comparable for the short and long versions [22,23]. The measurement invariance of these three scales are established as full strong among the German and Chinese students samples. In the present study, Cronbach’s alphas at baseline for depression were.890(Germany), and .776 (China). Alphas for anxiety were .795(Germany), and .737 (China). Alphas for stress were .851 (Germany), and .771 (China).

*Resilience*. Psychosocial stress resilience was assessed with an 11-item short version of the Wagnild and Young Resilience Scale (RS-14; 100; RS-11; 102) [24]. Participants responded to items such as “I usually manage one way or another” on a scale ranging from 1 (I disagree) to 7 (I agree). The RS-11 demonstrated good reliability and convergent validity in a German sample [25]. The measurement invariance of resilience is established as partial strong [16]. Cronbach’s alphas at baseline were .904(Germany), and .793 (China).

*Social Support*. Social support was assessed using the 14-item Questionnaire- Social Support measuring perceived and/or anticipated social support (F-SozU K-14) [26]. Participants indicated agreement with statements such as “I experience a lot of understanding and security from others” on a 5-point Likert scale ranging from 1 (*not true)* to 5 (*true*). In a German population, this unidimensional measure showed excellent Cronbach`s α and good convergent and discriminant validity [26]. In cross-cultural research, Nover (2012) [27] tested a long version of the Questionnaire- Social Support (F-SozU-22) [28] among pupils from Germany, Luxembourg and Spain, finding partial weak measurement invariance for the three cultural groups. The measurement invariance of the F-SozU K-14 is established as partial strong [16]. Cronbach’s alphas at baseline were .938 (Germany), and .948 (China).

*Social rhythm*. Social rhythm was assessed using the Brief Social Rhythm Scale (BSRS) [29]. This scale consists of ten items, which assess the irregularity with which participants engage in basic daily activities during the workweek and on the weekend. The BSRS assesses waking and bedtimes and breakfast and dinner mealtimes. It also assesses the regularity of time spent with others at work/school and during free time. Participants are asked to rate the general regularity of each activity in their lives in general using a scale ranging from 1 (very regularly) to 6 (very irregularly), with high mean scores indicating high irregularity. This measure can be administered at a single time point, rather than requiring a week of daily data to score. Summary scores are the average across all 10 items. The BSRS shows a slight positive skewed distribution. It is reliable, distinguishes among categories of mental health and detects relationships with physical and mental health, and is especially useful in large-scale or screening studies, where participant time is limited [29]. In the German representative telephone data, item-total correlations ranged from r=.25 to r=.54. Test-retest-reliability in a subsample study of 1294 people from Germany from the BOOM study who took the measure online or in paper and pencil format at time 1 and time 2 (4 weeks later) was r=.70. Cronbach’s alpha was α= .773 in Germany, and .837 in China. The measure was reversed scored in the present study so that high scores equal higher rhythmicity.

*Fertility wish*. The wish to have children (“Kinderwunsch” in German) was assessed using a single yes-no item asking “Do you want to have a child/children in the future?” Participants who already had a child, were asked “Do you want to have one more child / more children?” and responded indicating “no” or “yes” with how many children they wanted to have. Those answers were recoded into simple “no” or “yes.” In the Chinese student sample, no student had a child yet. In China, the legal age of marriage is 22 for male and 20 for female, which tends to lead to later childbearing. The ages correspond to 3rd or 4th year in university for males and 2nd or 3rd year in university for females. Our baseline sample in China is almost entirely freshmen and single, and none had children. In the German sample, 540 of total 591 responses to this question were from people who definitively had no existing child, and the rest may or may not have had a child already.

#### Sociodemographic predictors

*Basic sociodemographic predictors*. Sex, age, were assessed via self-report.

*Family affluence*. To ensure sufficient comparability across vastly different countries, the Family Affluence Scale (FAS) [30] served as the main cross-cultural measure of socioeconomic circumstances. The FAS is, a four-item measure of family wealth, developed in the WHO Health Behavior in School-aged Children Study. Questions include (either with 2 or 3 response alternatives): “Does your family own a car, van or truck?”, “Do you have your own bed- room for yourself?”, “During the past 12 months, how many times did you travel away on holiday with your family?”, and “How many computers does your family own?”. The FAS total score is calculated by summing up the responses to these items. Convergent validity is established via correlations with the Gross National Product across 35 countries [30]. Cronbach’s alphas at baseline were .355 (Germany), and .640 (China).

### Statistical analyses

The scales fulfilled the minimum requirement for path comparisons in cross cultural studies: weak invariance [16]. A series of structural equation models were conducted with all the predictors at baseline, predicting PMH outcomes at follow-up 1 and follow-up 2, to examine the relations between variables in this study. According to Kline [31], a minimum sample size for structural equation modeling (SEM) is about 200 cases. We have enough participants in Germany (591) and China (8,831) for SEM. Also, the present study is a follow up of the BOOM study [4], which is an exploratory study. Therefore, a sample size calculation was not conducted.

A multi group analysis was carried out to examine potential path equivalence between German and Chinese Students. The independence model, where paths vary freely, can be thought of as the baseline model (model 1, or M1). In this analysis, the full model was fit initially assuming invariance in the paths and covariances across the two countries (model 2, or M2). After the fit of this model was assessed, each pathways was individually evaluated for invariance across country by examining the critical ratio associated with the pathway when the pathway was freely estimated for the two countries separately. The model was then re-specified (model M2 partial, or M2p) a final time after including freely estimated coefficients for any pathways in which critical ratios exceeded conventional significance (p < .05). The fit of the less restrictive final model (Mp2) was then compared to the fully-restricted model and retained only if it appeared warranted by a statistically significant improvement in fit. All restrictive or partially restrictive models compare fit to the baseline independence model (M1).

The baseline independence model (M1), with no constraints on the paths will be tested in the first step. Model fit indices will be examined to assess the model’s fit. The root mean square of approximation (RMSEA) will be interpreted as follows: values above 0.10 indicate unacceptable fit [32], values in the range of 0.08 to 0.10 indicate mediocre fit, values between 0.05 and 0.08 indicate fair fit, and values less than 0.05 indicate close fit [33,34]. For the standardized root-mean-square residual (SRMR), values smaller than .09 indicate a good fit. Comparative fit index (CFI) [35] with values greater than .90, indicates a good fit. After M1 is established with at least acceptable Model fit indices, the same model with all paths being constrained as the same across the two countries (M2) will be tested and compared with M1. If M2 was not invariant between German and Chinese Students, the partially constrained model (M2p) will be tested. To test the partial invariant constrained model, paths were identified by means of modification indicies. At each step, the path with highest value of the modicifation index will be released and will be compared to M1. Since equality constraints will mostly lead to decreases in fit indices and the χ2 difference test is highly sensitive in large samples (Oishi, 2007), the rule of ΔCFI not greater than 0.01 [36] is recommended for model comparison. If ΔCFI between M1 and M2/M2p is not greater than 0.01, all constrained paths then can be seen as equal between German and Chinese Students. All analyses were calculated with SPSS 28 and R version 4.1.2 with the Package “SEM”.

Missing values were generally between .1 to 2.1%, depending on the measure, with Social Rhythm having 12.7% due to an error omitting 2 questions for some participants (missing at random with no statistical correlation to data). This had no statistical relationship to the results, and therefore participants with questionnaires with missing data relevant to the analyses were deleted from the analyses that involved those questionnaires. Internal consistency is computed with Cronbach’s α coefficient. Cronbach’s α > 0.70 indicates acceptable, > 0.80 good, and > 0.90 excellent internal consistency [37,38]. The standardized partial coefficients (betas) were calculated, with beta >=.10 for small effect, beta > = .30 for medium effect, and beta >= .50 for large effect according to Cohen (1988).

## Results

### Descriptive statistics

Table 1A presents data on participant demographics and descriptive statistics for the predictors and outcomes at Baseline and follow-ups. Both samples had a slightly larger number of female participants, with Germany having a higher percentage than China. Both samples reported a majority of students wishing to have children. The differences between Germany and China in gender and child wish were tested with chi-square test. Cramer’s V was calculated with V > .1 indicating small effect, V >.3 indicating medium effect, and V> .5 indicating large effect [38]. Gender showed a significant but tiny group effect with Cramer’s V = .036, which did not reach a small effect size with Cramer’s V > = .1 [38] and therefore can be ignored. Child wish showed no significant difference between Germany and China. The differences between Germany and China in age and FAS II were also examined via t-test. Hedge’s g was calculated with g > 0.2 indicating small effect, g > 0.5 indicating medium effect, and g > 0.8 indicating large effect [38]. The German students were significantly older than Chinese students (Hedges’ g = 2.21, with a large effect according to Cohen, p < .001) [38]. FAS II in German students was significantly higher than in Chinese students (Hedges’ g = .71, medium effect according to Cohen, p < .001) [38].

**Table 1:** a. Demographics and descriptive statistics for predictors and outcomes from participants with data at baseline and both follow ups. b. Demographics and descriptive statistics for predictors and outcomes from participants with data only at baseline, and comparisons with participants with data from all 3 waves.

	Germany		China	
	N	%	N	%
Gender				
Female	401	67.85%	5295	60.71%
Male	190	32.15%	3427	39.29%
Childwish BL				
Yes	472	87.41%	7881	89.42%
No	68	12.59%	932	10.58%
	Mean	SD	Mean	SD
Age BL	23.93	5.15	19.52	1.58
FASII BL	4.03	1.85	2.56	2.08
Social rhythm BL	41.02	7.80	43.51	7.99
Resilience BL	58.37	11.36	58.73	8.37
Social support BL	60.23	10.04	56.85	11.92
Stress BL	7.04	4.67	3.24	3.05
Anxeity BL	2.88	3.39	2.81	2.63
Depression BL	4.21	4.41	1.74	2.36
Positive mental health BL	18.85	5.73	21.25	4.93
Positive mental health FU1	18.56	5.92	19.95	5.25
Positive mental health FU2	17.84	6.05	20.15	5.30

	Germany			China		
	N	%	Crames’ V	N	%	Crames’ V
Gender			.053***			.048***
Female	4638	57.47%		2909	65.64%	
Male	3432	42.53%		1523	34.36%	
Childwish BL			.014			.044***
Yes	5836	85.53%		4087	86.48%	
No	987	14.47%		639	13.52%	
	Mean	SD	Hedges’ g	Mean	SD	Hedges’ g
Age BL	23.74	4.70	.040	20.11	2.24	.327***
FASII BL	3.94	1.84	.045	3.23	2.24	.312***
Social rhythm BL	40.79	8.43	.027	43.86	7.93	.044*
Resilience BL	56.97	12.12	.116**	57.86	8.67	.103***
Social support BL	59.13	10.74	.103**	56.43	12.18	.035*
Stress BL	7.41	4.71	.079*	3.62	3.27	.122***
Anxiety BL	3.35	3.59	.131**	3.17	2.95	.133***
Depression BL	4.64	4.54	.095*	2.04	2.67	.120***
Positive mental health BL	18.15	5.73	.123***	20.85	5.28	.080***

Note: BL = Baseline, FU1 = Follow-Up1, FU2 = Follow-Up2, social rhythm with higher score = more regularity.

Note: BL = Baseline

* = p<.05

** = p<.01

*** = p<.001.

Table 1B presents data on participant demographics and descriptive statistics for the predictors and outcomes at Baseline from 2011 to 2016 as well as the results from chi-square tests with effect size Crames’s V and t-tests with effect size Hedges’ g, which compared the difference between participants who took part in all three waves and those who only took part in baseline wave. In Germany, no significant difference, which reached a small effect size, was found. In China, age and FASII at baseline with small effects were found to be significantly different between those who took part in all three waves and who only took art in baseline wave. Chinese students, who was older at baseline and or with higher score of FASII, were more likely to drop out.

#### Correlations

The correlations among the psychological predictors are shown in Table 2. The salutogenic predictors (positive mental health, resilience, social support, social rhythm regularity, and child wish) at baseline are generally positively correlated with positive mental health at Follow-Ups. The baseline negative aspects of mental health, or pathogenic predictors (i.e., stress, anxiety, and depression) are negatively correlated with positive mental health at Follow-Up1 and Follow-Up2.

**Table 2:** Correlations among the psychological predictors within country.

	Control		Baseline pathogenic predictors			Baseline salutogenic predictors				Outcomes at follow-ups	
	Family Affluence	Age	Stress	Anxiety	Depression	Social Support	Resilience	Positive Mental Health	Social rhythm	Positive Mental Health FU1	Positive Mental Health FU2
**China**											
Family Affluence in BL	1										
Age in BL	-.132**	1									
Stress in BL	-.005	-.065**	1								
Anxiety in BL	.007	-.079**	.722**	1							
Depression in BL	-.018	-.031**	.658**	.662**	1						
Social Support in BL	.161**	-.078**	-.164**	-.151**	-.224**	1					
Resilience in BL	.061**	.054**	-.323**	-.322**	-.388**	.299**	1				
Positive Mental Health in BL	.077**	.044**	-.482**	-.458**	-.528**	.341**	.546**	1			
Social rhythm in BL	.132**	.116**	-.293**	-.271**	-.270**	.160**	.362**	.361**	1		
Positive Mental Health FU1	.067**	.036**	-.310**	-.290**	-.305**	.202**	.308**	.434**	.239**	1	
Positive Mental Health FU2	.101**	.066**	-.248**	-.239**	-.260**	.178**	.277**	.388**	.215**	.418**	1
**Germany**											
Family Affluence in BL	1										
Age in BL	-.137**	1									
Stress in BL	-.096*	.057	1								
Anxiety in BL	-.045	-.017	.567**	1							
Depression in BL	-.079	.046	.593**	.598**	1						
Social Support in BL	.113**	-.036	-.294**	-.333**	-.506**	1					
Resilience in BL	.021	-.053	-.205**	-.221**	-.345**	.309**	1				
Positive Mental Health in BL	.107**	-.05	-.537**	-.455**	-.669**	.562**	.476**	1			
Social rhythm in BL	.125**	-.078	-.251**	-.251**	-.337**	.296**	.187**	.294**	1		
Positive Mental Health FU1	.114**	-.041	-.423**	-.393**	-.522**	.468**	.349**	.698**	.262**	1	
Positive Mental Health FU2	.186**	-.075	-.423**	-.365**	-.481**	.458**	.316**	.639**	.279**	.700**	1

* = p<.05

** = p<.01

***=p<.001.

#### Structural equation model

Results indicated that the initial fully constrained M2 model provided an inadequate overall fit. The chi-square was statistically significant, χ^2^ (23) =111.689, p < .001, and the RMSEA=.032, SRMR=.010) indicated inadequate fit compared to the perfectly fitting independence model (M1), as indicated by the ΔCFI = .021, which exceeded .01.

**Fig 1:**
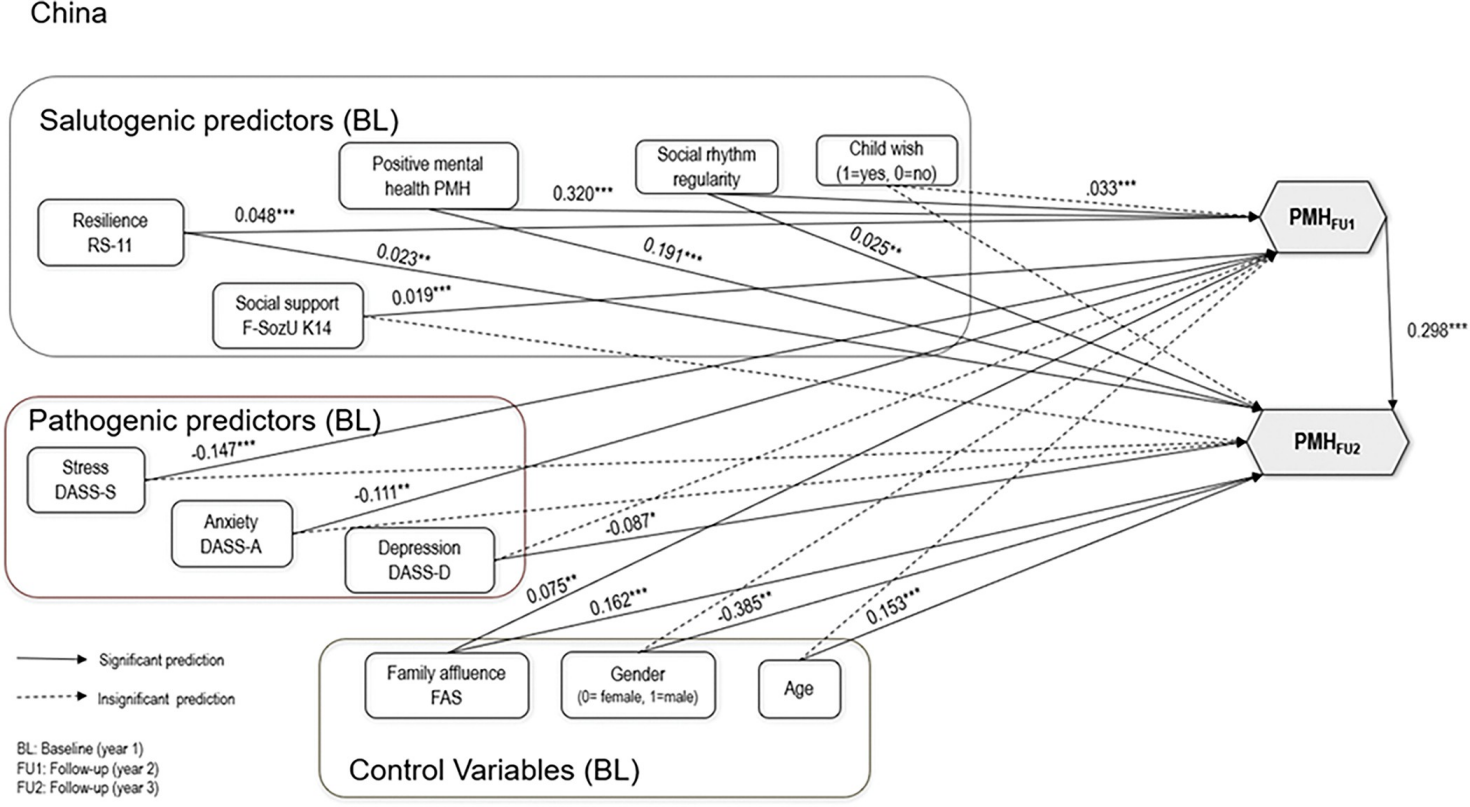
Baseline Structural Equation Model (M1) to Predict Positive Mental Health in China.

**Fig 2:**
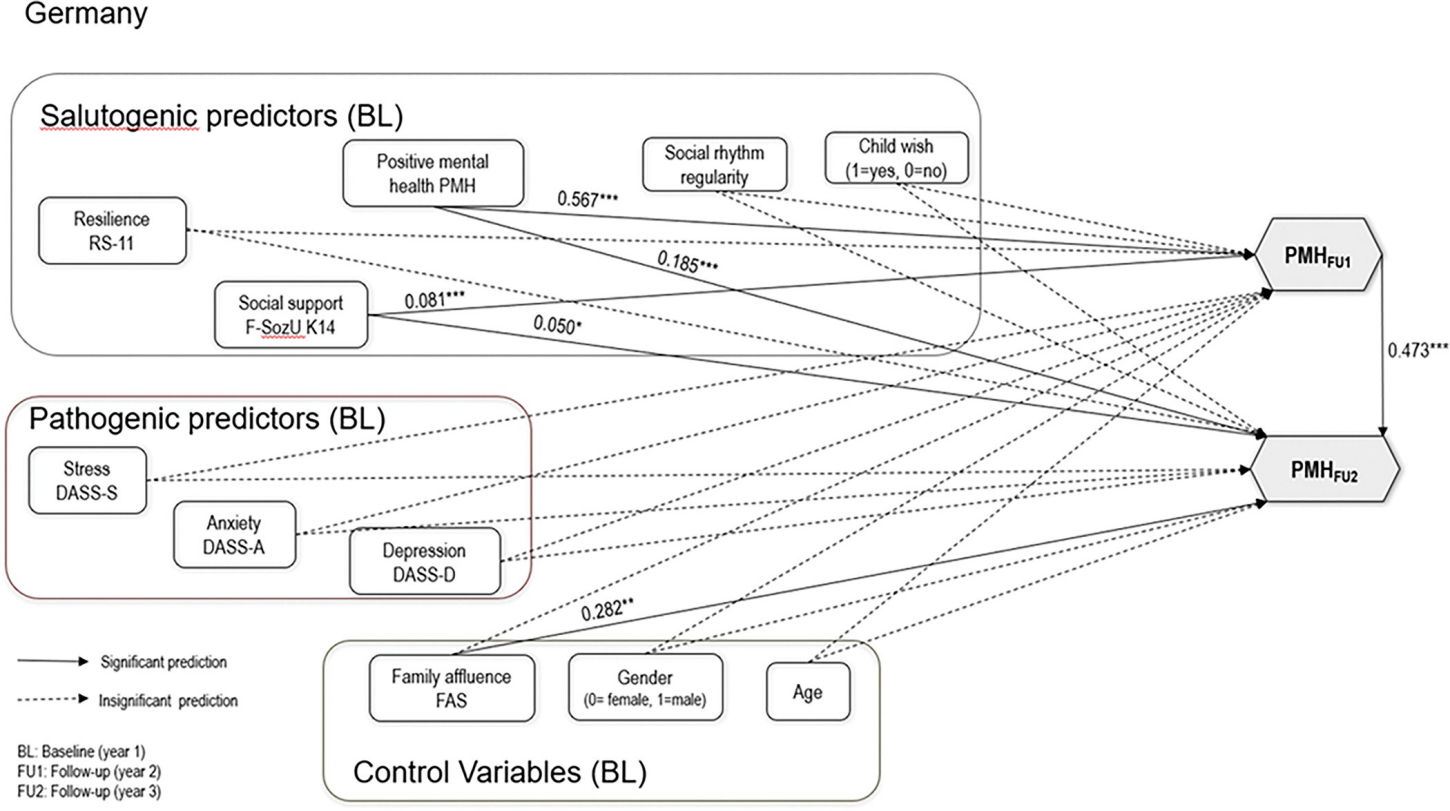
Baseline Structural Equation Model (M1) to Predict Positive Mental Health in Germany.

Nonetheless, inspection of the individual pathways of even this strictly constrained reference model did reveal that, consistent with past findings, positive mental health at baseline significantly predicted positive mental health at follow-ups (see Figs 1 and 2). Figs 1 and 2 depict the standardized path and covariance estimates from the model, in which links among baseline salutogeneic, pathogeneic, and control predictors are linked to follow-up 1 and follow-up 2 PMH in Chinese and German students. Pathways depicted with solid lines were statistically significant at p = .05 or greater. Predicted pathways that were not significant are depicted with dashes. For clarity, error estimates for measured and latent variables are not displayed.

**Fig 3:**
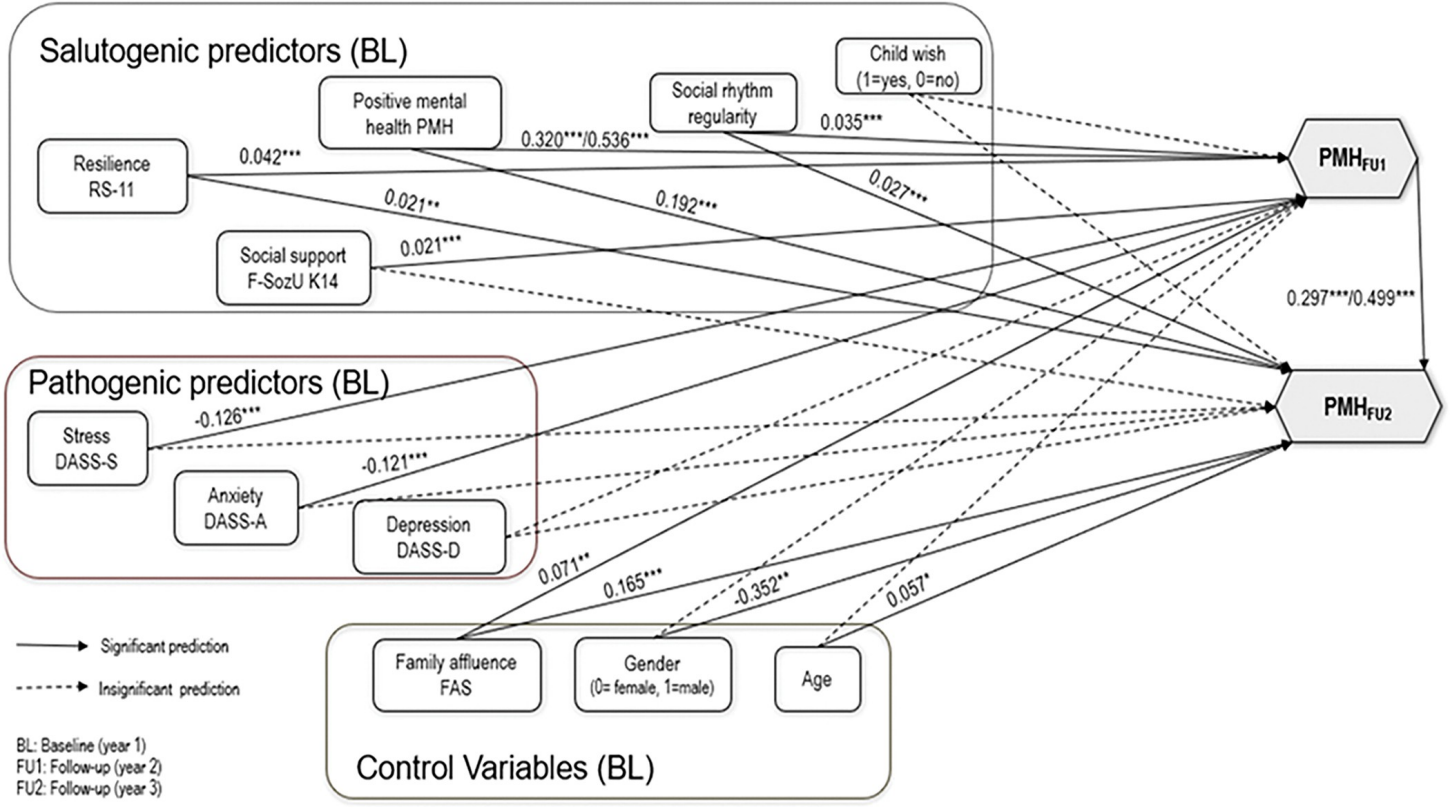
Partial constrained model with two Unconstrained Paths to Predict Positive Mental Health in China and Germany. Note: Unconstrained paths are the paths from PMH baseline to PMH FU1, PMH FU1 to PMH FU2. The coefficients were presented as: China/Germany.

Because of the CFI changes of the M2 model, we conducted further examination of the partially constrained model between countries. These indicated that the assumption of country invariance was untenable in two specific instances: namely, the path from PMH at baseline to PMH at follow-up 1, and the path from PMH at follow-up 1 to follow-up 2. The fit of the less restrictive model (M2p) in which these pathways was estimated separately for each country was χ^2^(21) = 45.196, p = .001), Δχ^2^(21) =45.196, p =.001, when comparing M2p to M1. The comparative fit was CFI = .994. RMSEA for the model was .017 and the SRMR was .004. After allowing these two paths to be freely estimated between the countries, ΔCFI=.006, did not exceed .01, when comparing M2p to M1. Thus, this partially constrained model (M2p) can be considered equal to the baseline independence M1 model in terms of CFI changes. As it is recommended that the most restrictive model that still fits the data be used in interpretation, we present and interpret M2p as the final model in Fig 3, with two coefficients for China and Germany given separately for the two unconstrained pathways. Table 3 presents the standardized path coefficients from all structural equation models. Table 4 provides the model fit indices for the unconstrained model (M1) as well as the full constrained model (M2) and the partially constrained model (M2p). In the baseline, independence model M1, regression weights were estimated for the both countries completely separately.

**Table 3:** Standardized regression coefficients (betas) from structural equation models.

	M1				M2		M2p	
	Positive mental health FU1		Positive mental health FU2		Positive mental health FU1	Positive mental health FU2	Positive mental health FU1	Positive mental health FU2
	Germany	China	Germany	China	Germany/China	Germany/China	Germany/China	Germany/China
**R^2^**	.512	.216	.542	.238	.362/.228	.355/.253	.504/.215	.531/.237
Age BL	-.065	.015	-.083	.153***	-.012	.052	-.006	.057*
Gender	.713	.101	-.011	-.385**	.165	-.328**	.142	-.352**
Childwish BL	-.512	.336	.273	.17	.296	.194	.284	.202
FASII BL	.024	.075**	.282**	.162***	.073**	.165***	.071**	.165***
Social rhythm BL	.007	.035	.021	.025**	.033***	.025**	.035***	.027***
Positive mental health BL	.567***	.320***	.185**	.191***	.341***	.195***	.536***.326***	.192***
Resilience BL	.011	.048***	.01	.023**	.043***	.021**	.042***	.021**
Social support BL	.081**	.019***	.050*	.006	.021***	.007	.021***	.007
Stress BL	-.033	-.147***	-.082	-.000	-.131***	-.025	-.126***	-.024
Anxiety BL	-.102	-.111**	-.082	-.055	-.106**	-.046	-.121***	-.056
Depression BL	-.024	-.021	.042	-.087*	-.059	-.084**	-.024	-.057
Positive mental health FU1	-	-	.473***	.298***	-	.312***	-	.499***/.297***

Note.

* = p<.05

** = p<.01

***=p<.001.

M1=Baseline Model, M2= fully constrained Model, M2p= partial constrained Model.

**Table 4:** Fit indices for the unconstrained and constrained models.

	χ^2^	df	CFI	RMSEA	SRMR	Δχ^2^	Δdf	p	ΔCFI
M1									
Germany	0	0	1.000	.000	.000				
China	0	0	1.001	.000	.000				
Baseline Model M1	0	0	1.000	.000	.000				
M2	111.689	23	.979	.032	.010	111.689	23	<.001	.021
M2 partial	45.196	21	.994	.017	.004	45.196	21	.001	.006

The results of the model M1 indicated that in China students’ sample, two of the six salutogenic predictors (PMH with beta = .320/.191 indicating medium/small effect, resilience with beta = .048/.023 indicating small effects) at baseline were predictive of PMH at both Follow-Ups (FU1/FU2). Social support at baseline was only predictive of PMH at Follow-Up 1 with beta = .019 indicating small effect. Social rhythm regularity at baseline was only predictive of PMH at Follow-Up 2. Pathogenic predictors stress (beta = -.147, indicating small effect) and anxiety (beta = -.111, indicating small effect) were predictive of PMH at Follow-Up 1, but not of PMH at Follow-Up 2. Depression at baseline (beta =-.087, indicating small effect)was predictive of PMH at Follow-Up 2, but not of PMH at Follow-Up 1. The control variable family affluence (beta = .075/.162, indicating small effects) was positively related to PMH at both Follow-Ups (FU1/FU2). Being male (beta = -.385, indicating medium effect) in this study was associated with decreased PMH at Follow-Up 2. Age (beta = .153, indicating small effect) was positively related to PMH at Follow-Up 2.

In German students’ sample, two of the six salutogenic predictors (PMH with beta = .567/.185, indicating large/small effect), social support with beta = .081/.050, indicating small effects) at baseline were predictive of PMH at both Follow-Ups (FU1/FU2). Pathogenic predictors were not predictive of PMH at both Follow-Ups. The control variable family affluence (beta = .282, indicating small to medium effect) was positively related to PMH at Follow-Up 2.

The results of model M2p indicated overall positive effects of every positive baseline predictor (PMH, family affluence, social rhythm, resilience, and social support) on follow-up mental health except childwish, and negative effects of every negative baseline predictor (stress, anxiety, depression). Two paths were significantly different between China and Germany: the path from baseline PMH to FU1 PMH as well as the path from FU1 PMH to FU2 PMH. The effects of baseline PMH on FU1 PMH were stronger in Germany than in China. In predicting FU2 PMH, baseline age, PMH, family affluence, social rhythm, and resilience were positive predictors. FU1 PMH was also a positive predictor of FU2 PMH, again with the effect stronger in Germany than in China. None of the negative predictors (stress, anxiety, or depression) were significant predictors of FU2 PMH, nor were baseline childwish or social support. Gender was a predictor of FU2 PMH, in that being female (coded as 0) predicted higher FU2 PMH than being male (coded as 1).The standardized partial coefficients (betas) were presented in Table 3, with beta >=.10 indicating small effect, beta > = .30 indicating medium effect, and beta >= .50 indicating large effect according to Cohen (1988).

## Discussion

The present study is a three time-point follow-up to our prior, two-time point cross-cultural, prospective study using the BOOM data [4], and examining the the prediction of positive and negative mental health over time, from a range of psychological salutogenic and pathogenic predictors across countries. The present study focuses in on the prediction of positive mental health, extending findings across three time points, in Germany and China (three time points were not available in the dataset from Russia), and extends earlier findings [4] by providing evidence that salutogenic factors not only predict future PMH over an extended period of time, but also that salutogenic factors may be more likely to predict positive future mental health than pathogenic variables.

In our prior study examining effects across three countries (Germany, China, and Russia) and two time points, results could be considered consistent across the three countries. Six salutogenic predictors were examined, including somatic health, social support, resilience, PMH, social rhythm regularity, and fertility wish. These were all predictive of both PMH (positively). Five of the salutogenic factors (all except resilience) were related negatively to depression. Although pathogenic predictors anxiety and depression were also related to future depression, they were not significant predictors of future PMH after taking baseline salutogenic factors into account [4]. The findings of the present study indicated some variation by country. First, there were some mean differences, in that people from China reported higher social rhythm, resilience, and PMH, and lower stress, anxiety, and depression, as well as social support. This trend toward greater positive and lower negative mental health in Chinese participants was also reported in our prior study [4]. Mean comparisons were not within scope of the present study, but an examination and comparison of mental health across these two cultures should be further explored. Indeed other recent studies have found that differences between students from China and Germany on measures of mental health are not significant [39].

Models indicated that in Chinese students, three of the six salutogenic predictors (social rhythm regularity, PMH, resilience) at baseline were predictive of PMH at both follow-ups. Social support at baseline was only predictive of PMH at follow-up 1. This is a change from the first study, where social support, somatic health, and fertility wish were additional significant predictors [4]. In addition, in the present study, stress and anxiety were predictive of PMH at follow-up 1, but not of PMH at follow-up 2, while depression at baseline was predictive of PMH at follow-up 2, but not of PMH at follow-up 1. In the prior study, negative mental health wasn’t significantly related to later PMH [4]. It may be that these small effects are not reliable enough to consistently produce significant results. As in the prior study [4], and consistent with expectations set by prior research [9,40], family affluence was also positively related to PMH at both follow-ups. Being male in this study was associated with decreased PMH at follow-up 2. Age was positively related to PMH at follow-up 2.

For German students, two of the six salutogenic predictors (PMH, social support) at baseline were predictive of PMH at both follow-ups. This is in contrast to the prior study, where all six salutogenic predictors were predictive of PMH at the first follow-up [4]. Pathogenic predictors were not predictive of PMH at either of the two follow-ups, consistent with our prior two-point study [4]. Family affluence was again positively related to PMH at follow-up 2.

The utility of social support and social rhythm regularity in predicting PMH have firm grounding in prior research [9,11,29,41,42], and our results are consistent with the prior findings. Perceived social support serves as a protective factor for mental health [43]. It reduces mental illness incidence, general stress in both women and men [44], and has beneficial effects on physical health [45]. Social rhythm, the regularity with which one engages in social activities, and is related to PMH [29,46,47]. While our prior study indicated fertility wish (a potential sign of optimism about the future) as a significant predictor of PMH [4], this effect did not hold up in this three-point study. Those who have children live longer than those who don’t in Western society [48], but more research needs to be done on the desire to have children and its potential reflection or even effect on mental and physical health. The findings regarding resilience and its prediction of PMH in China are consistent with prior research on the importance of resilience in PMH [49], making the lack of significance in the German sample in this study surprising.

In the partially constrained model, the German and Chinese pathways were constrained to be equal, except for the pathway from PMH at baseline to PMH at follow up 1, and from PMH at follow up 1 to PMH at follow-up 2. In both cases, the relationship was stronger in Germany than in China. Otherwise, the pattern of results and significance generally followed the pattern seen in the separate model for China. This is likely due to the much larger sample size in China (N = 8,831) than in Germany (N = 591). The sample was heavily weighted toward China, resulting in not much change in the strength of effects when adding the German data and constraining them to be equal. In some cases, however, the German effect sizes were similar to those in China, yet were only significant in China, again likely due to sample size. For example, the effect of baseline anxiety of follow-up 1 PMH was -.102, ns, in Germany, but -.111, p<.01, in China. When combined, the effect was .121, p<.001. While the partially constrained model was adopted due to it being the most concise model that fit the data, it may still be useful to examine the data separately, given the large difference in sample size, and the possibility that some of the German effects or lack thereof may be essentially swallowed up by combining the German and Chinese data. For example, the German effects of social rhythm, family affluence, and resilience were very small and nonsignificant, but when they were combined with the Chinese data, the combined effects resembled the larger and significant Chinese effects. Also, the effect of social support was larger for the German students, but became smaller (though still significant) when combined with the Chinese data.

An examination of the effects over time, generally indicated smaller effects of baseline variables on follow-up 2 PMH than on follow-up 1 PMH. This generally makes sense, as FU2 is more distal and effects tend to regress toward the mean over time. However, there were two exceptions. Social rhythm had a strengthened effect at time 2, suggesting that keeping and maintaining a good social rhythm has an addtitive effect on PMH. Also, childwish had a stronger negative effect on time two PMH. We had not considered this possibility, finding in the contemporaneous data from the current Chinese and German samples that childwish is associated with some aspects of health, particularly for Chinese students, including quality of health, happiness (Chinese men and women) and satisfaction with life (Chinese women), as well as lower depression (German men) possibly due to it being a stand in for feeling secure and healthy enough to have a child [50]. However, unfulfilled childwish may have a negative impact on mental health over time, as it may go unfulfilled [51]. Even if a person’s wish to have a child is fulfilled, research indicates that having a child, while a meaningful experience, can also bring on significant stress, which may contribute to worsening PMH over time [52–54].

The findings speak to the importance of resilience, and extend past findings beyond the typical North American studies, across Euro and Asian cultures, to Germany and China. The results are consistent with the prior examination of this data across two time points. Results from the prior study indicated that in China, Germany, and Russia, neary all of the included salutogenic predictors (somatic health, social support, resilience, PMH, social rhythm regularity, and fertility wish), were predictive of both PMH and negatively predictive of negative mental health, as measured by depression [4]. One salutogenic factor was not related to later depression (resilience). Pathogenic predictors anxiety and depression were related to future depression, but, anxiety and depression were not significant predictors of later PMH in the model with basline salutogeneic factors included. Baseline stress was related to later PMH (negatively) and depression (positively), in the expected directions.

The present study has a number of strengths, including the large sample size, particularly in the Chinese student data, and its breadth of measures and longitudinal nature. Another strength of the present study is the use of cross-cultural samples outside the North American norm. The present study also has limitations. First, there was a large discrepancy in sample size between the German and Chinese samples, and combining the data in the constrained model, may have simple subsumed the German effects. A comparable sample of German students would have provided a more balanced model on which to test the data constraints. Second, it would have been interesting to include pathogenic outcomes for comparison, but this was beyond the scope of this study. Third, the study used university student samples, which are not representative of the population, and thus generalizability is limited. Fourth, it would have been useful to includ clinical sub-samples in this study, to account for more of the full range of depression and anxiety symptoms. Fifth, as one reviewer noted, the values reported from China were generally lower than those reported from German sample. However, in a few cases, values were higher in Chinese than in German students; namely in the domains of social rhythm, resilience, and PMH. This led the reviewer to concerns about systematic measurement bias, and the meaningfulness of comparing path coefficients. However, as measurement invariance was obtained, the comparisons of path coefficients were meaningful, and thought not to be caused by systematic measurement bias. Any possible differences caused by method could be examined with latent mean comparisons, but is not within scope of the present study. Participants and dropouts did not differ on the main study measures in meaningful ways. Finally, although the model provided a good fit to the data, the effect sizes were small, and the model did not explain a large amount of variance in the data as a whole. This would suggest that this remains an incomplete model. Specifically, the R^2^ values reported in Table 3 range from 0.216 to 0.512. The reviewer pondered the clinical relevance of a prediction model that fails to capture even half of the variability. Studies that try to explain human behavior generally have R^2^ values less than 50%. Human behavior is simply harder to predict than physical processes and multiply-determined. However, even a tiny R^2^=.001 can be meaningful, as long as the effect under investigation can unfold over time [55].

In sum, results indicate prediction of PMH over an extended period of time, and in particular by salutogenic predictors. Pathogenic predictors were (negatively) predictive of PMH only in China, underscoring the differential prediction of PMH from salutogenic and pathogenic factors. However, some of the non significant effects in Germany, may be attributed to the smaller sample size in Germany. Future studies should include similar sample sizes in both countries, and compare outcomes for PMH to those for negative mental health. PMH aspects should be considered more thoroughly in both creating resilience against mental disorder and contributing to optimal healthy flourishing [56].

## Supporting information

S1 Appendix (DOCX)

S1 File (SAV)
