# Supplementary material for: Three-wave longitudinal prediction of positive mental health in Germany and China
Source: PLoS One. 2023 Dec 21;18(12):e0287012. doi: 10.1371/journal.pone.0287012 (PMC10735011; doi:10.1371/journal.pone.0287012)
Supplement: S1 Appendix — (DOCX) [file pone.0287012.s001.docx]

Appendix 1

Data transparency statement

This work is part of The BOOM Studies, a multi-national, longitudinal research study examining mental health across cultures and time. Other publications have arisen from this study, but do not replicate the analyses conducted in this paper. Data have been made available as Supplemental Information
